# Bipolar Covalent Organic Frameworks Crafting Core–Shell Heterostructured Cathode for High–Performance Lithium–Organic Batteries

**DOI:** 10.1002/advs.75241

**Published:** 2026-04-15

**Authors:** Yalong Jiang, Nan Jiang, Yu Dou, Zixuan Chen, Yunhai Zhu, Long Chen, Yingkui Yang

**Affiliations:** ^1^ State Key Laboratory of New Textile Materials and Advanced Processing Wuhan Textile University Wuhan China; ^2^ School of Chemistry and Materials Science South–Central Minzu University Wuhan China; ^3^ State Key Laboratory of Supramolecular Structure and Materials College of Chemistry Jilin University Changchun China

**Keywords:** bipolar organics, carbon nanotube, covalent organic framework, hierarchical structure, lithium–organic batteries

## Abstract

Covalent organic frameworks (COFs) with tunable molecular structures and ordered porous channels have emerged as promising cathode materials for lithium–organic batteries. However, their practical application is still hindered by low operating voltages, limited specific capacities, and sluggish charge transport kinetics. In this work, a new carbon nanotube@bipolar COF (CNT@COF) core–shell heterostructure cathode was rationally crafted through the synergistic integration of molecular and microstructural engineering. The robust 3D hybrid architecture features a conductive CNT core and COF shells enriched with p–/n–type nitrogen electroactive sites, enabling rapid electron/ion transport while maximizing electrochemical utilization. Consequently, the CNT@COF cathode exhibits a high reversible capacity of 226.5 mAh g^−1^, outstanding rate capability (137 mAh g^−1^ at 5 A g^−1^) and long–term cycling stability (82.5% at 5 A g^−1^ over 5000 cycles), outperforming most previously reported COF–based organic cathodes. The charge storage mechanism is further revealed by ex situ X–ray photoelectron spectroscopy, operando Fourier transform infrared spectroscopy, and theoretical calculations, involving PF_6_^−^ anions interacting with C–N bonds during p–type oxidation and Li^+^ cations binding to C = N bonds through n–type reduction. Moreover, the assembled symmetric batteries exhibit both high–energy and high–power densities and operate effectively even under low–temperature conditions.

## Graphical abstract

A bipolar CNT@COF core–shell heterostructure cathode constructed via synergistic molecular and microstructural engineering enables rapid electron/ion transport and efficient utilization of electroactive groups for high–performance lithium–organic batteries.

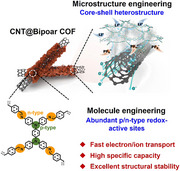

## 1 Introduction

Lithium–ion batteries (LIBs) have emerged as the dominant technology in consumer electronics, electric vehicles, and stationary energy storage over the past few decades [1]. Accordingly, extensive efforts have been dedicated to developing advanced electrode materials to further enhance their electrochemical performance. For inorganic cathode materials, the rising costs of key raw materials such as lithium and cobalt, coupled with environmental concerns associated with their extraction and processing, have spurred the search for sustainable alternatives. Organic materials have emerged as promising candidates owing to their eco–friendliness, resource abundance, renewability, and highly tunable synthetic versatility [2, 3, 4, 5, 6]. Their structural diversity allows for the precise design of a wide range of redox–active materials. Nevertheless, most organic materials still suffer from intrinsic drawbacks, including a low density of active sites, poor electronic conductivity, and dissolution in electrolytes, which collectively result in low specific capacity, inferior rate capability, and poor cycling stability. In contrast, polymeric materials offer distinct advantages, as their molecular architectures can be rationally tailored through monomer selection, enabling controlled insolubility and optimized electrochemical performance.

Covalent organic frameworks (COFs), a class of crystalline and porous polymers, offer significant advantages for electrochemical energy storage owing to their extended π–conjugated and robust backbones, ordered pore architectures, and structural tunability, which allows for the precise incorporation of electroactive groups into their frameworks [7, 8, 9, 10]. The electrochemical performance of COFs is largely governed by the nature of these electroactive groups, which are generally classified as n–type or p–type based on their redox characteristics. Owing to their higher theoretical capacity and diverse structural motifs (e.g., C═O and C═N), most COF–based cathodes reported to date belong to the n–type category. However, their relatively low redox potentials limit the achievable energy density [11, 12, 13]. In contrast, p–type groups such as tetraphenyl–p–phenylenediamine, phenoxazine, and aromatic amines can deliver higher redox potentials (> 3.5 V vs. Li/Li^+^), but are typically constrained by limited specific capacities [14, 15]. To combine the high capacity of n–type and the high voltage of p–type groups, a molecular engineering strategy that introduces both p– and n–type electroactive groups into a single framework to construct bipolar COFs represents a promising route toward cathodes with simultaneously high capacity and high operating voltage. Beyond electroactive groups, intrinsic structural features of COFs also impose performance limitations. Strong π–π interactions often lead to tightly packed parallel stacking, which restricts ion accessibility to inner electroactive sites, thereby reducing capacity and slowing ion diffusion kinetics. Moreover, the intrinsically poor electrical conductivity of most COFs further hinders charge–transfer kinetics [16]. To address these issues, integrating COFs with conductive carbonaceous materials such as carbon nanotubes [11, 17, 18, 19] and graphene [20, 21] has emerged as an effective strategy. These conductive scaffolds not only enhance electron transport but also mitigate structural degradation during repeated ion insertion/extraction processes due to their excellent mechanical resilience. Accordingly, there is an urgent need to develop carbon–composite bipolar COF cathode materials that simultaneously deliver high specific capacity and fast charge–transfer kinetics. Achieving this goal requires synergistic advances in both molecule–level design (e.g., rational placement of electroactive groups) and microstructure–level engineering (e.g., heterostructure construction), which remains a highly promising yet underexplored research direction.

**FIGURE 1:**
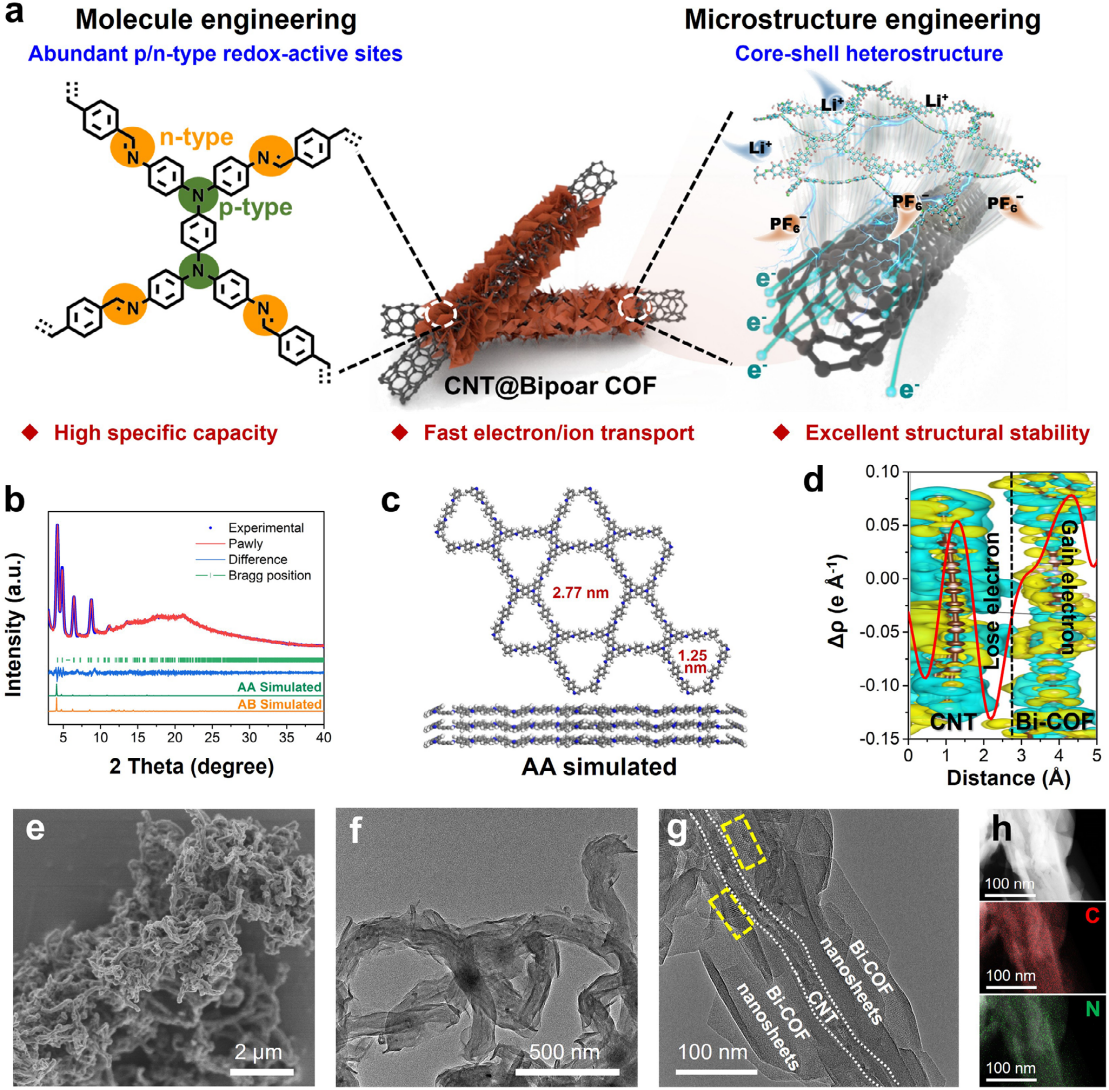
(a) Schematic illustration of the redox–bipolar CNT@COF core–shell heterostructure designed at both molecular and microstructural levels, achieving high specific capacity, fast electron/ion transport, and excellent structural stability. (b) XRD pattern fitting of Bi–COF with Pawley refinement. (c) Structural unit of Bi–COF derived with the AA–stacking model. (d) Parallel view of the charge density difference image and the planar average potential difference versus the transverse direction of the CNT@COF heterostructure. (e–h) SEM, TEM, HRTEM images, and EDS mappings of CNT@COF–3.

Herein, we report a carbon nanotube@bipolar covalent organic framework (CNT@COF) core–shell heterostructure as a bipolar cathode through the synergistic integration of molecular and microstructural engineering strategies. As illustrated in Figure 1a, the incorporation of abundant p–type sp^3^ nitrogen centers and n–type C = N linkages, coupled with the fast ion/electron transport enabled by the conductive CNT core and porous COF nanosheet shell, endows the CNT@COF electrode with high specific capacity, excellent rate capability, and outstanding cycling stability, surpassing the performance of most previously reported COF–based organic cathodes. Furthermore, the charge storage mechanism is systematically investigated using ex situ X–ray photoelectron spectroscopy (XPS), operando Fourier transform infrared spectroscopy (FTIR), and density functional theory (DFT) calculations. Finally, the symmetric LIBs assembled with CNT@COF electrodes demonstrate both high energy and power densities, along with stable operation under low–temperature conditions.

## 2 Results and Discussion

### 2.1 Synthesis and Characterization of Bipolar CNT@COF

First, bipolar COF (Bi–COF) was synthesized through a one–step Schiff–base condensation reaction of TA and TP in a mixture of n–butyl alcohol/o–dichlorobenzene/aqueous acetic acid at 120°C with a yield of 90%. The CNT@COF composites were prepared by incorporating 5, 10, and 20 wt.% of CNT using the same method, named CNT@COF–X (X = 1, 2, 3). Elemental analysis confirms that the C, H, and N contents of Bi–COF were close to the theoretical values (Table S1). To further demonstrate the successful synthesis of Bi–COF and CNT@COF, a series of structural and morphological characterizations was conducted. In the FTIR spectra of the as–obtained samples (Figure S1), the peak observed at 1616 cm^−1^ is associated with the stretching vibration of C = N, which indicates the formation of an imide bond [22]. Additionally, the other two absorptions at 1265 and 1315 cm^−1^ correspond to the symmetric and asymmetric vibrations of the C–N bond in the TP unit [23]. The crystal structures of Bi–COF and CNT@COF were investigated using X‐ray Diffraction (XRD) measurement combined with structural simulation. As shown in Figure 1b, Bi–COF displays diffraction peaks at 2.38°, 4.16°, 4.79°, 6.36°, and 8.69°, which are assigned to the Bragg diffractions of (100), (110), (200), (210), and (300) crystal planes, respectively. The simulated AA and AB stacking models represent a dual–pore framework with Kagome lattices featuring eclipse and staggered stacking (Figure 1c; Figure S2). The simulated XRD pattern of the AA stacking model aligns well with the experimental result of Bi–COF, with the converged Rwp and Rp values of 3.61% and 2.75%. This demonstrates that the crystalline Bi–COF belongs to the space group of P1 (a = 42.1 Å, b = 42.47 Å, c = 4.22 Å, α = β = 90°, γ = 119.14°). As the CNT content increases, the position of the diffraction peaks remains unchanged, but the degree of crystallization decreases (Figure S3). The solid–state ^13^C NMR spectrum provides additional details on the chemical structure of Bi–COF (Figure S4). Chemical shifts at 145.0 and 156.4 ppm correspond to the carbon atom adjacent to the nitrogen atom of the TP units and imine linkages with the characteristic C = N resonances. N_2_ adsorption–desorption experiments were conducted to investigate the pore size distribution and specific surface area of the obtained samples (Figure S5). Bi–COF and CNT@COF–1, 2, and 3 exhibit BET surface areas of 987, 326, 285, and 184 m^2^ g^−1^, respectively. Their adsorption isotherms combine characteristics of both type I and IV with pore sizes of mesopore (2.52 nm) and micropore (1.27 nm), which are consistent with the theoretical hexagonal (2.77 nm) and triangular (1.25 nm) pores of the AA stacking model. The chemical bonds in CNT@COF–3 were further investigated by XPS measurements. The N 1s XPS spectra exhibit two peaks at 398.9 and 399.7 eV, attributed to the C = N and C–N bonds in Bi–COF (Figure S6) [11]. To explore the role of core–shell heterostructure on electron transport, the differential charge density of CNT@COF heterostructure was calculated using DFT calculations (Figure 1d; Figure S7). The yellow and green colors represent the areas of electron accumulation and attenuation, respectively. The pronounced partial charge delocalization of Bi–COF due to π–π stacking and the electron transfer between Bi–COF and CNT suggest that the CNT@COF heterostructure exhibits efficient charge transport and superior structural stability. Additionally, the planar average potential difference indicates a charge transfer from CNT to Bi–COF (Figure 1d). Thermogravimetric analysis (TGA) results demonstrate that the Bi–COF and CNT@COF are thermally stable up to 470°C in a nitrogen atmosphere, indicating an outstanding thermal stability (Figure S8). The content of CNT in CNT@COF–1, 2, and 3 is calculated as 17.7, 31.2, and 50.9 wt.%, respectively, exceeding their corresponding initial feed ratios. This discrepancy is likely attributed to the negative effect of CNTs on the yield of the COF. FTIR and XRD results of Bi–COF remain unchanged after immersion in different organic solvents (N, N–dimethylformamide, dimethyl sulfoxide, ethanol, and N–methyl–2–pyrrolidone) and water for three days at room temperature, demonstrating the good chemical stability (Figure S9). The morphology of Bi–COF and CNT@COF was characterized by scanning electron microscope (SEM) and transmission electron microscope (TEM). Bi–COF displays a microflower structure composed of nanosheets (Figure S10), while the CNT@COF composites present the unique core–shell heterostructure owing to the intense π–π interaction between COF and CNT during the polymerization process (Figure 1e, f; Figures S11 and S12). Figure 1g displays the high–resolution TEM (HRTEM) images of CNT@COF–3, clearly revealing the CNT core and COF nanosheet shell, thereby confirming the formation of the heterostructure. Additionally, the lattice fringes of the Bi–COF (inset in Figure S10c) and CNT@COF–3 (inset in Figure 1g), highlighted by yellow dashed boxes, indicate the crystallinity of Bi–COF. The energy‐dispersive X‐ray spectroscopy (EDS) mappings of Bi–COF (Figure S10d) and CNT@COF–3 (Figure 1h) demonstrate a uniform distribution of C and N elements. The high–angle annular dark–field scanning transmission electron microscopy (HAADF–STEM) imaging combined with line–scan EDS analysis was performed across individual CNTs (Figure S13). The EDS line profiles exhibit a pronounced increase in C intensity in the central region, consistent with the presence of the CNT core. These results collectively verify that the CNT@COF composite possesses a well–defined core–shell architecture, where CNTs act as the conductive core and the COF constitutes the outer shell. Additionally, statistical analysis revealed that the average thickness of the COF nanosheets in the CNT@COF–3 heterostructure is approximately 79.4 nm (Figure S14).

### 2.2 Electrochemical Performance of Lithium–Organic Half Cells

**FIGURE 2:**
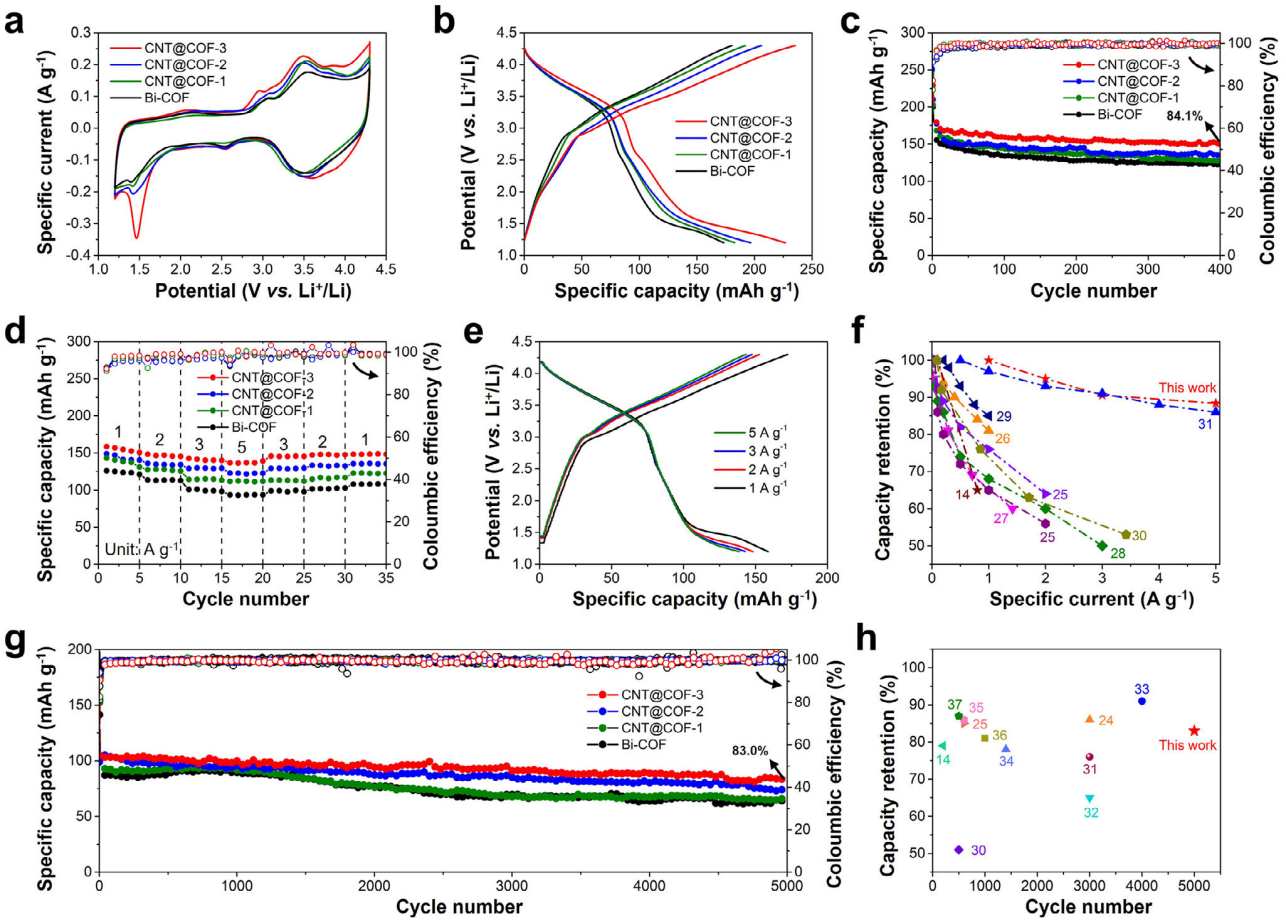
Electrochemical performance of Bi–COF and CNT@COF electrodes. (a) CV curves of Bi–COF and CNT@COF at a scan rate of 0.5 mV s^−1^. (b) Galvanostatic charge/discharge curves at 200 mA g^−1^. (c) Cycling performance of Bi–COF and CNT@COF at 500 mA g^−1^ for 400 cycles. (d) Rate performance of Bi–COF and CNT@COF. (e) Galvanostatic charge/discharge curves of CNT@COF–3 at different current densities. (f) Comparison of CNT@COF–3 and other COF–based cathodes in terms of rate performance. (g) Cycling performance of Bi–COF and CNT@COF at 5 A g^−1^ for 5000 cycles. (h) Comparison of CNT@COF–3 with other reported COF–based cathodes in terms of cycling performance.

The cyclic voltammetry (CV) measurements were conducted at a scan rate of 0.5 mV s^−1^ within the voltage range of 1.2–4.3 V (vs. Li/Li^+^) (Figure 2a). The two pairs of redox peaks observed between 2.8 and 4.3 V (vs. Li/Li^+^) correspond to the reversible doping process of PF_6_^−^ ions at the C–N active centers of triphenylamine units, while the redox peaks between 1.2 and 2.8 V (vs. Li/Li^+^) relate to the reversible insertion and extraction of Li^+^ ions at redox–active C = N groups. Figure 2b displays the galvanostatic charge–discharge (GCD) curves of Bi–COF and CNT@COF at 200 mA g^−1^. CNT@COF–3 delivers a specific capacity of 226.5 mAh g^−1^, which is higher than that of Bi–COF (173.2 mAh g^−1^), CNT@COF–1 (182.3 mAh g^−1^), and CNT@COF–2 (196.5 mAh g^−1^). This improvement is attributed to the formation of CNT@COF core–shell heterostructure, which facilitates electron and ion transport within the electrode materials and increases the utilization of active sites. Moreover, the increased CNT content improves the proportion of core–shell heterostructures within CNT@COF, further enhancing the specific capacity. The CNT electrode delivers a reversible specific capacity of ∼12 mAh g^−1^ at 200 mA g^−1^ (Figure S15), indicating that the capacity contribution from CNTs in the CNT@COF electrode is minimal. CNT@COF–3 delivers a specific capacity of 149.2 mAh g^−1^ with a capacity retention of 84.1% at 500 mA g^−1^ after 400 cycles, which is higher than that of Bi–COF (122.7 mAh g^−1^ with 76.3%), CNT@COF–1 (127.6 mAh g^−1^ with 75.5%), and CNT@COF–2 (136.2 mAh g^−1^ with 78.1%) (Figure 2c). The rate performance of Bi–COF and CNT@COF electrodes was evaluated (Figure 2d). CNT@COF–3 displays superior rate capability with reversible capacities of 154, 147, 141, and 137 mAh g^−1^ at current densities of 1, 2, 3, and 5 A g^−1^, respectively. The reversible specific capacity of CNT@COF–3 recovers to 148 mAh g^−1^ when the current density is restored to 1 A g^−1^, indicating excellent reversibility of the redox reactions. The GCD curves of CNT@COF–3 at different current densities remain consistent without significant polarization, indicating superior reaction kinetics (Figure 2e). Benefiting from the rapid reaction kinetics, the CNT@COF electrode exhibits excellent rate performance in comparison to other reported COF–based organic cathodes (Figure 2f, Table S2) [14, 25, 26, 27, 28, 29, 30, 31]. Figure 2g displays the long–term cycling performance of the obtained electrodes at 5 A g^−1^. CNT@COF–3 delivers a notable specific capacity of 85.1 mAh g^−1^ after 5000 cycles with a capacity retention ratio of 83.0%. The exceptional cycling performance is attributed to the robust heterostructure framework of CNT@COF, which possesses strong chemical resistance against the dissolution of intermediates in the electrolyte and mitigates structural degradation caused by ion absorption/desorption due to the excellent mechanical strength of the CNT core. Compared to previously reported COF–based cathodes, the CNT@COF electrode exhibits significantly enhanced cycling stability (Figure 2h and Table S3) [14, 24, 25, 30, 31, 32, 33, 34, 35, 36, 37].

### 2.3 Kinetic Analysis

**FIGURE 3:**
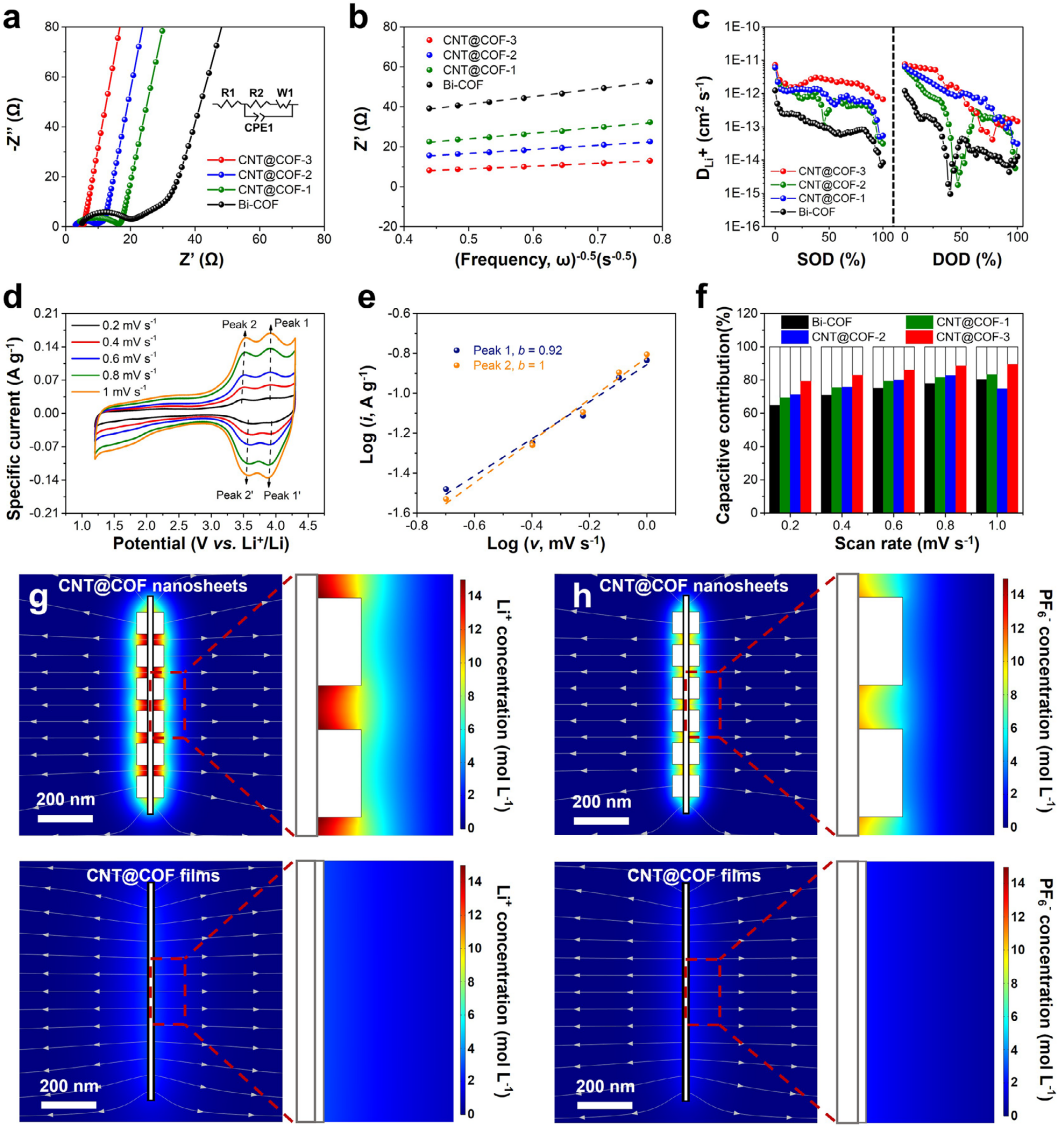
Kinetics analysis of Bi–COF and CNT@COF electrodes. (a) EIS curves with applied inset equivalent circuit. (b) The relationship between the real part of impedance (Z’) and the reciprocal of the square root of frequency (ω^−1/2^). (c) The ionic diffusivities based on GITT. (d) CV curves of CNT@COF–3 at scan rates from 0.2 to 1.0 mV s^−1^. (e) Log (v) versus log (i) plots of Peak 1 and Peak 2 of CNT@COF–3. (f) Contribution ratio of the capacitive–controlled processes at various scan rates. Simulated concentration and flux distribution of (g) Li^+^ and (h) PF_6_^−^ ions around CNT@COF nanosheets and CNT@COF films.

The electrochemical reaction kinetics were initially studied using electrochemical impedance spectroscopy (EIS) measurements. As depicted in Figure 3a, the Nyquist plots consist of a semicircle in the high–to–medium frequency range representing the charge–transfer resistance (Rct), and an inclined line in the low–frequency range associated with the Li^+^ diffusion ability defined as the Warburg impedance (σ). The Rct value of CNT@COF–3 (5 Ω) is lower than that of Bi–COF (21 Ω), CNT@COFc1 (17 Ω), and CNT@COF–2 (9 Ω) determined by fitting the equivalent circuit (inset in Figure 3a), which is advantageous for the charge transfer process. Additionally, CNT@COF–3 exhibits the lowest diffusion resistance σ, indicating rapid ion transport within the heterostructure framework (Figure 3b). The galvanostatic intermittent titration technique (GITT) measurements were also performed to quantify the ion diffusion process (Figure S16). The calculated ion diffusion coefficient of CNT@COF–3 falls within the range of about 10^−14^ to 10^−11^ cm^2^ S^−1^ during the charge/discharge process, higher than that of other electrode materials (Figure 3c), contributing to the improved rate performance. To further investigate the reaction kinetics, CV curves were recorded at scan rates ranging from 0.2 to 1 mV s^−1^. The CV curves of Bi–COF and COF@CNT electrodes display similar shapes with paired redox peaks, denoted as Peak 1, 1’ and Peak 2, 2’ (Figure 3d and Figure S17). The relationship between the scan rate (ν) and peak current (i) is generally described by the equation: *i = a v^b^* [38], where the b value typically falls within the range of 0.5 to 1. The b value of 0.5 suggests a predominantly diffusion–controlled reaction process, while b value approaching 1 indicates that the reaction process is primarily governed by capacitive behavior. As shown in Figure 3e and Figure S18, the b values of Peak 1 and Peak 2 of COF@CNT–3 are 0.92 and 1, respectively, which are higher than those of other materials, indicating a capacitance–dominated characteristic. Furthermore, the contributions of capacitive and diffusion–controlled processes were quantified using the following equation: *i* = *k*_1_ *v* + *k*_2_ *v* ^1/2^ [38], where *k*_1_ *v* and *k*_2_ *v* ^1/2^ correspond to the capacitive and diffusion–controlled contributions, respectively. As the scan rate increases, the capacitive contribution of all electrode materials also rises (Figure 3f). For COF@CNT–3, the capacitive contribution increases from 79.2% at 0.2 to 89.4% at 1 mV s^−1^, exceeding that of other electrodes. This high capacitive contribution indicates the superior reaction kinetics of COF@CNT heterostructures, consistent with the previous kinetic analysis results. Finite element simulations were performed using COMSOL Multiphysics to investigate the concentration fields of Li^+^ and PF_6_^−^ ions in different hierarchical structures. To ensure computational efficiency while retaining relevance, the spatial configuration of CNT@COF nanosheets was simplified into a 2D model, where vertically aligned nanoarrays are distributed on a rectangular substrate. For comparison, a separate model representing a film–like COF–coated CNT structure was also constructed. As illustrated in Figure 3g, h, the nanoconfinement effect within the vertical arrays promotes the accumulation of Li^+^ and PF_6_^−^ ions under the influence of an electric field, thereby enhancing ion transport and enabling superior high–rate performance. In contrast, the film–coated structure fails to effectively concentrate ion effectively near the electrode surface, underscoring the structural advantages of the core–shell heterostructures.

### 2.4 Redox Reaction Mechanism of Bipolar CNT@COF

**FIGURE 4:**
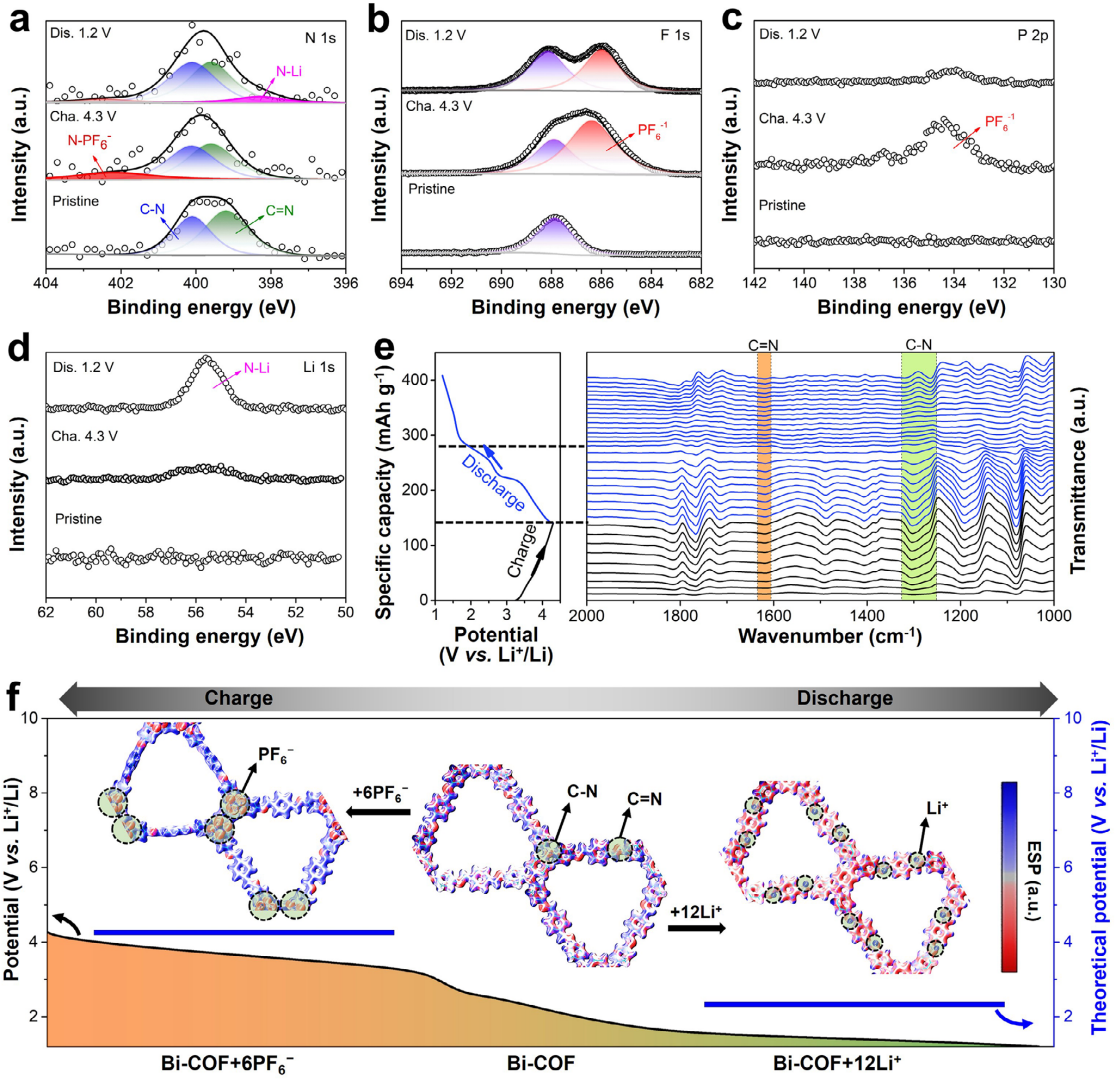
Electrochemical reaction mechanism characterizations. Ex situ (a) N 1s, (b) F 1s, (c) P 2p, and (d) Li 1s XPS spectra of CNT@COF–3 at various charge/discharge states during the first cycle. (e) Operando FTIR and corresponding charge and discharge curve of CNT@COF–3 during the first cycle at 200 mA g^−1^. (f) Electrostatic potential distributions of Bi–COF, Bi–COF+6PF_6_^−^, and Bi–COF+12Li^+^, and theoretical potential calculations.

The XPS measurements during the first cycle were initially conducted to gain insights into the redox reaction mechanism of the CNT@COF electrode. The high–resolution N 1s XPS spectra of the fresh CNT@COF–3 electrode could be deconvoluted into two peaks, corresponding to C–N bonds of TP units and the C = N linkage (Figure 4a). Upon charging, peaks at 684.9 eV in F 1s XPS spectra (Figure 4b) and 134.4 eV in P 2p XPS spectra (Figure 4c) emerge for CNT@COF–3, corresponding to PF_6_^−^ anions, indicating their adsorption on the CNT@COF electrode. Meanwhile, a new peak located at 402.2 eV appears in N 1s XPS spectra during charging (Figure 4a), attributed to the interaction between the positively charged N^+^ in TP units and PF_6_^−^ anions, resulting in the formation of a stable N–PF_6_^−^ complex during charging [22]. After discharging, new peaks emerge at 398.3 eV in N 1s XPS spectra (Figure 4a) and 55.6 eV in Li 1s XPS spectra (Figure 4d) of CNT@COF–3. These peaks are attributed to the reduction process wherein the n–type redox–active C = N sites preferentially gain electrons while simultaneously binding Li^+^ ions, leading to the formation of a C–N–Li interaction to maintain electrical neutrality [39]. To further explore the changes in C–N and C = N bonds within CNT@COF during the charging and discharging process, operando FTIR spectra were recorded during the first cycle (Figure 4e). The characteristic peak of the C–N single bond at 1268 cm^−1^ is downward and then gradually intensifies during the charging process, indicating continuous consumption of the C–N bond due to the interaction of PF_6_^−^ anions with the C–N bonds in TP units. Upon discharging to approximately 2.2 V (vs. Li/Li^+^), a reverse process occurs, indicating the gradual restoration of the C–N bonds with the desorption of PF_6_^−^ anions. Upon further discharging to 1.2 V (vs. Li/Li^+^), the intensification of the downward peak of C = N double bonds and the upward peak of C–N bonds suggests that the C = N double bonds are reacting with Li^+^ cations to form C–N–Li. This observation is consistent with the ex situ XPS analysis results. Additionally, the DFT calculations were conducted to investigate the rational redox–active sites of Bi–COF. The molecular surface electrostatic potential of Bi–COF was calculated (the middle one in Figure 4f), revealing that the nitrogen element exhibits a lower electrostatic potential due to molecular conjugation, with the local electrostatic potential at the center of the C = N bond being lower than that of the C─–N bond. This suggests that the n–type redox–active C = N sites preferentially gain electrons and the C–N bond tends to become positively charged, allowing it to bind with PF_6_^−^ anions as a p–type active site. Additionally, a theoretical model was developed based on the electrochemical capacity of Bi–COF at different potential ranges, wherein a single Bi–COF repeating unit incorporates 6 PF_6_^−^ anions during charging and 12 Li^+^ ions after discharging. The geometry optimization of Bi–COF at three states was conducted (Figure S19). The planar structure of Bi–COF undergoes greater deformation when interacting with PF_6_^−^ anions compared to binding with Li ions due to the higher charge density of PF_6_^−^ anions. Furthermore, the theoretical redox potentials of Bi–COF at different charge/discharge states were calculated (Figure 4f). The calculated redox potentials of 2.63 and 4.28 V (vs. Li/Li^+^) closely match the experimentally measured values, confirming the interaction of PF_6_^−^ anions with p–type oxidation in the 3–4.3 V (vs. Li/Li^+^) range and the binding of Li^+^ ions with n–type reduction in the 1.2–3 V (vs. Li/Li^+^) range. These theoretical results elucidate the p/n–type charge storage mechanism of the Bi–COF electrode, highlighting its potential for application in symmetric LIBs.

### 2.5 Electrochemical Performance of Symmetric Lithium–Organic Batteries

**FIGURE 5:**
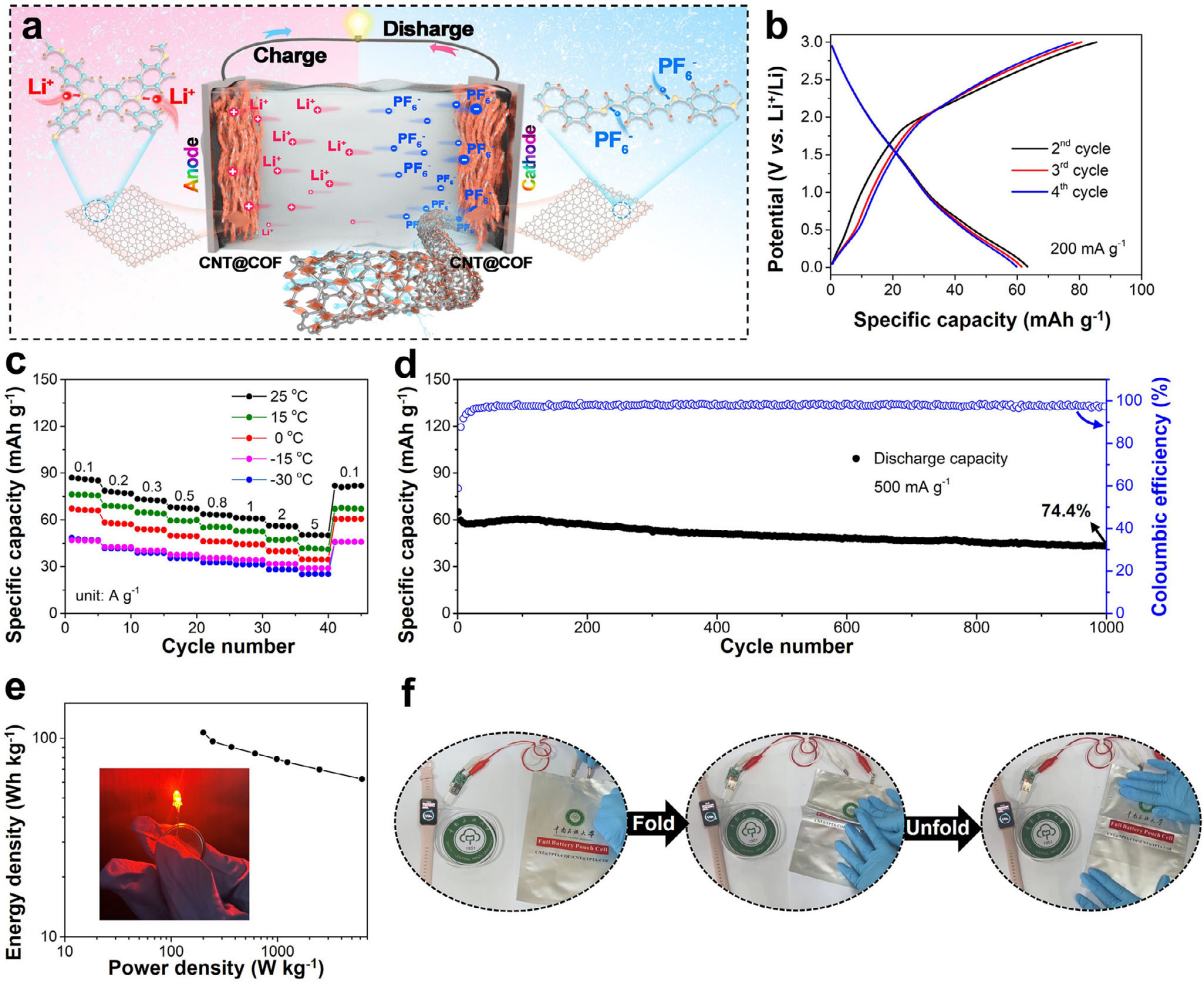
Electrochemical performance of symmetric LIBs based on the CNT@COF–3 electrode. (a) Schematic illustration of a symmetric LIB utilizing redox–active C = N center for Li^+^ ions storage at the anode and C–N center for PF_6_^−^ storage at the cathode. (b) GCD curves at 200 mA g^−1^. (c) Rate performance under different temperatures. (d) Long–term cycling performance at 500 mA g^−1^. (e) Ragone plot of energy density versus power density plot (a single coin cell can power an LED light). (f) Photographs of an electronic watch powered by a pouch cell during the folding and unfolding process, demonstrating flexibility and functionality.

Given the substantial potential difference of about 2.0 V between the oxidation and reduction potentials, combined with the excellent electrochemical performance of the CNT@COF electrode, it is proposed to construct symmetric LIBs using CNT@COF as both the anode and cathode. As illustrated in Figure 5a, the symmetric LIBs operate by binding Li^+^ ions to the redox–active C = N center at the anode, while PF_6_^−^ anions interact with the C–N center at the cathode during redox reactions. Figure 5b displays the GCD curves of the symmetric LIBs at 200 mA g^−1^ between 0.01 and 3 V, showing an average output voltage of approximately 1.9 V and a high specific capacity of 74.9 mAh g^−1^. The symmetric LIBs exhibit a superior rate performance at room temperature of 25°C (86.1, 77.6, 72.7, 67.6, 63.3, 61, 56, and 50.2 mAh g^−1^ at current densities from 0.1 to 5 A g^−1^) (Figure 5c). Notably, when the current density is reduced back to 100 mA g^−1^, the specific capacity recovers to 81.5 mAh g^−1^, highlighting the excellent capacity recoverability. Besides, maintaining the superior electrochemical performance of energy storage devices at low temperature conditions is crucial for practical applications. The symmetric LIBs show only a slight decline in specific capacity as the temperature decreases, with specific capacities of 50.3, 41.7, 34.6, 28.9, and 25.1 mAh g^−1^ at 5 A g^−1^ under 25, 15, 0, –15, and –30°C, respectively (Figure 5c). Long–term cycling stability of symmetric LIBs was evaluated at 500 mA g^−1^ for 1000 cycles (Figure 5d). A specific capacity 43.3 mAh g^−1^ with a capacity retention of 74.4% was achieved after 1000 cycles, demonstrating excellent cycling stability. The symmetric LIBs achieve a specific energy of 106 Wh kg^−1^ at 200 W kg^−1^, with a high power density of 6232 W kg^−1^ at an energy density of 62 Wh kg^−1^ (Figure 5e). The capability of the symmetric LIB is further demonstrated by successfully powering an orange light–emitting diode (LED) light (inset in Figure 5e). In summary, the symmetric LIBs exhibit excellent electrochemical performance even at low temperatures, offering valuable insights into the development of low–temperature all–organic batteries. Finally, a flexible symmetric LIBs pouch cell based on CNT@COF electrode was assembled using aluminum–plastic packaging films. Impressively, the electronic watch continued to charge during the folding and unfolding process of the cell (Figure 5f; Supporting Video), highlighting the potential for flexible wearable devices.

## 3 Conclusion

In summary, we successfully synthesized a bipolar CNT@COF core–shell heterostructure cathode through the synergistic integration of molecular and microstructural engineering. The integration of the highly conductive CNTs core and COF shell enriched in p/n–type nitrogen redox sites enables rapid electron/ion transport and efficient utilization of electroactive groups. Consequently, the optimized CNT@COF composites exhibit a high reversible capacity of 226.5 mAh g^−1^, excellent rate capability (137 mAh g^−1^ at 5 A g^−1^) and long–term cycling stability (82.5% at 5 A g^−1^ after 5000 cycles), outperforming most reported COF–based organic cathodes. Additionally, the charge storage mechanism was investigated using ex situ XPS, operando FTIR, and theoretical calculations, involving PF_6_^−^ anions interacting with the C–N bonds during p–type oxidation (3–4.3 V vs. Li/Li^+^), while Li^+^ cations binding to C = N bonds during n–type reduction to form C–N–Li species (1.2–3 V vs. Li/Li^+^). Kinetics analysis further confirms the capacitance–dominated behavior of CNT@COF composite characterized by low charge–transfer resistance and fast ion diffusion. Importantly, symmetric LIBs assembled with CNT@COF exhibit stable electrochemical performance even at a low temperature of −30°C, highlighting the potential for practical applications. This work provides valuable insights into the rational design of organic electrodes with both high capacity and enhanced reaction kinetics for next–generation lithium–organic batteries.

## Conflicts of Interest

The authors declare no conflicts of interest.

## Supporting information




**Supporting File 1**: advs75241‐sup‐0001‐SuppMat.docx.

**Supporting File 2**: advs75241‐sup‐0002‐Movie S1.mp4.

## Data Availability

The data that support the findings of this study are available from the corresponding author upon reasonable request.
