# Supplementary material for: Bipolar Covalent Organic Frameworks Crafting Core–Shell Heterostructured Cathode for High–Performance Lithium–Organic Batteries
Source: Adv Sci (Weinh). 2026 Apr 15:e75241. Online ahead of print. doi: 10.1002/advs.75241 (PMC13334607; doi:10.1002/advs.75241)
Supplement: Supplementary file 1 — Supporting File 1: advs75241‐sup‐0001‐SuppMat.docx. [file ADVS-9999-e75241-s002.docx]

Supporting Information

**Bipolar Covalent Organic Frameworks Crafting Core-Shell Heterostructured Cathode for High-Performance Lithium-Organic Batteries**

Yalong Jiang^1^, Nan Jiang^2^, Yu Dou^2^, Zixuan Chen^1^, Yunhai Zhu^1^, Long Chen^3^* and Yingkui Yang^1^*

^1^ State Key Laboratory of New Textile Materials and Advanced Processing, Wuhan Textile University, Wuhan 430200, China

^2^ School of Chemistry and Materials Science, South-Central Minzu University, Wuhan 430074, China

^3^ State Key Laboratory of Supramolecular Structure and Materials, College of Chemistry, Jilin University, Changchun 130012, China

* Corresponding author: longchen@jlu.edu.cn (L.C.), ykyang@wtu.edu.cn (Y.Y.)

**Experimental Methods**

**Synthesis of bipolar COF (Bi-COF) and CNT@COF**: Typically, Bi-COF was synthesized via the polycondensation reactions. In brief, N, N, N′, N′-tetraphenyl-1,4-phenylenediamine (TP) (23.63 mg, 0.05 mmol) and terephthalaldehyde (TA) (13.41 mg, 0.10 mmol) were dissolved in a mixture of o-dichlorobenzene/n-butyl alcohol/3 M aqueous acetic acid (5:5:1 v/v/v, 2.2 mL). After sonication for 30 minutes, the solvothermal reaction was performed at 120 ^o^C for five days. The product was then isolated by vacuum filtration, washed with dimethylacetamide (DMAc) and tetrahydrofuran, and dried at 100 ^o^C under reduced pressure for 12 h to obtain Bi-COF materials with a yield of 90%. The CNT@COF-1 composite was synthesized following a similar procedure with the addition of multi-wall carbon nanotube (CNT). Specifically, 1.5 mg of acidified CNT was firstly dispersed into a mixture of o-dichlorobenzene/n-butyl alcohol/3 M aqueous acetic acid (5:5:1 v/v/v, 2.2 mL) and ultrasonicated for 30 minutes. TP and TA were then added to the dispersion followed by another 30-min sonication. The solvothermal reaction was performed at 120 ^o^C for five days. Finally, the product was isolated by vacuum filtration, washed with DMAc and tetrahydrofuran and dried at 100 ^o^C for 24 h. CNT@COF-2 and CNT@COF-3 composites were synthesized using the same method with 3 mg and 6 mg of CNTs, respectively.

**Materials Characterization**: Powder X-ray diffraction (XRD) measurements were carried out with a Rigaku Miniflex 600 system using Cu/Kα radiation (λ=0.1542 nm). The scan speed was 5° min^−1^ with a step size of 0.0025°. The Pawley refinement of the experimental XRD data was conducted using Materials Studio 7.0 software. Nitrogen adsorption/desorption isotherms were measured to investigate the specific surface area and pore size distribution of samples with an ASAP 2020 PLUS HD88 instrument after degassing under vacuum at 150 °C for 12 h. The chemical structure was performed by Fourier transform infrared spectroscopy (FTIR; NEXUS 470) spectra within the wavenumber range of 4000–500 cm^−1^. 13C CP solid-state nuclear magnetic resonance (NMR) spectra were collected on a Bruker NEO 400MHz NMR spectrometer. X-ray photoelectron spectroscopy (XPS) was conducted on a Thermo Multi Lab 2000 spectrometer. The thermal stability was studied by thermogravimetric analysis (TGA; TG209 F3) under nitrogen atmosphere with a heating rate of 10 °C min^−1^ between 40 and 700 °C. The surface morphology was observed by scanning electron microscope (SEM; SU 8010) and transmission electron microscope (TEM; Hitachi HT7700).

**Electrochemical measurements**: The working electrodes were prepared by mixing 60 wt.% active materials, 30 wt.% Ketjen black (conductive carbon) and 10 wt.% polyvinylidene fluoride (PVDF) in N-methyl-2-pyrrolidone (NMP) to form a homogeneous slurry. The resulting slurry was cast onto an Al foil current collector, dried at 80 ^o^C under vacuum overnight, and then punched into circular electrode discs with a diameter of 10 mm. The half cells were assembled with the working electrodes as the cathode, Li foil as the counter electrode, 1 M LiPF_6_ in ethylene carbonate (EC)/ethyl methyl carbonate (EMC) (3:7 v/v) as the electrolyte, and a glass-fiber membrane (Celgard 2500) as separator. The loading mass of cathodes on the Al foil was about 0.6-0.8 mg cm^−2^. All-organic full cells were fabricated with the fresh CNT@COF-3 used as both cathode and anode. The fabrication of coin-type and pouch-type cell followed the same procedure as the half-cells without pre-treatment. Electrochemical measurements were conducted using CR2032-type coin cells assembled in an Ar-filled glovebox (H_2_O/O_2_ < 0.1 ppm). The galvanostatic charge/discharge tests were performed on a LAND CT2001A (China) battery testing system at room temperature. Cyclic voltammetry (CV) and electrochemical impedance spectra (EIS, 0.1 Hz–100 kHz frequency) measurements were recorded on the CHI 660E (China) electrochemical workstation.

**Galvanostatic Intermittence Titration Technique (GITT)**: The Li-ion diffusion coefficient (D) can be estimated depend on Fick’s second law, which is following simplified formula Equation:

$$D=\frac{4}{\pi\tau}\left( \frac{m_{B}V_{M}}{M_{B}S} \right)^{2}\left( \frac{{\Delta E}_{S}}{{\Delta E}_{\tau}} \right)^{2}$$

Where *τ* is the relaxation time, *M_B_*, *S*, *m_B_* and *V_M_* are the molar mass, electrode-electrolyte interface area, mass and molar volume of electrode material, respectively. *ΔE_S_* and *ΔE_τ_* are voltage drops, which can get from the charge/discharge curves.

**Ex-situ XPS spectroscopy spectrum**: The ex-situ XPS measurements were conducted based on the electrode after cycling in half cells and then disassembled at the specific potential point. After washing with 1,3-Dioxolane (DOL) for several times in the argon-filled glove box to remove the electrolyte, the electrodes were dried under vacuum at room temperature before the measurements.

**Computational details**: All the calculations were performed in the framework of the density functional theory with the projector augmented plane-wave method, as implemented in the Vienna ab initio simulation package (VASP). The generalzied gradient approximation proposed by Perdew, Burke, and Ernzerhof was selected for the exchange-correlation potential. The long-range van der Waals interaction was described by the DFT-D3 approach. The cut-off energy for plane wave was set as 500 eV. In the structural optimization calculations, the thresholds of self-consistent field energy convergence and residual forces on atoms were respectively set as 1 × 10^−5^ eV and -2 × 10^−2^ eV Å^−1^, as well as the Г-centred k-point mesh was sampled by the gamma scheme with a separation of 0.04 Å^−1^. In the static calculations, the threshold of self-consistent field energy convergence was set as 1 × 10^−8^ eV and the Г-centred k-point mesh was sampled by the gamma scheme with a separation of 0.02 Å^−1^, whereby the accurate Gibbs free energies and electronic structures could be subsequently acquired. The visualization of electrostatic potentials was acquired by processing to the calculation results through the software of Multiwfn and Visual Molecular Dynamics (VMD). The crystal structure and charge density difference were visualized by the software of VESTA.


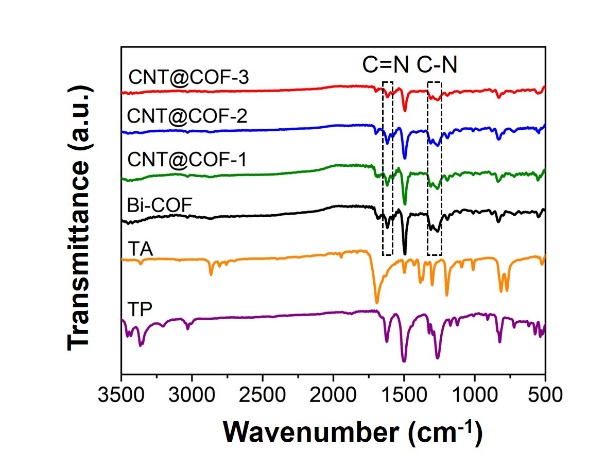


**Figure S1.** FTIR spectra of TP, TA, Bi-COF and CNT@COF composites.


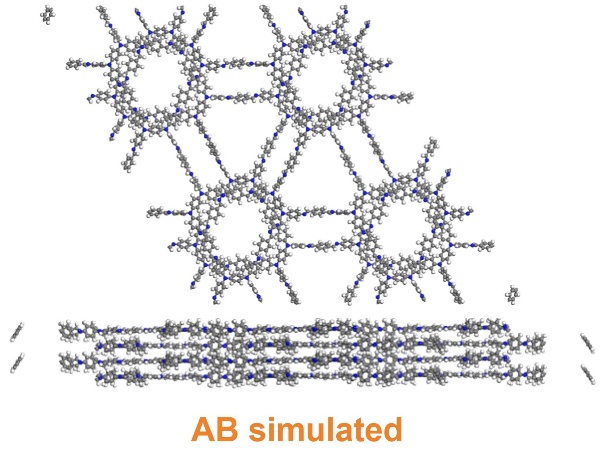


**Figure S2.** Structural unit of the Bi-COF derived with the AB-stacking models.


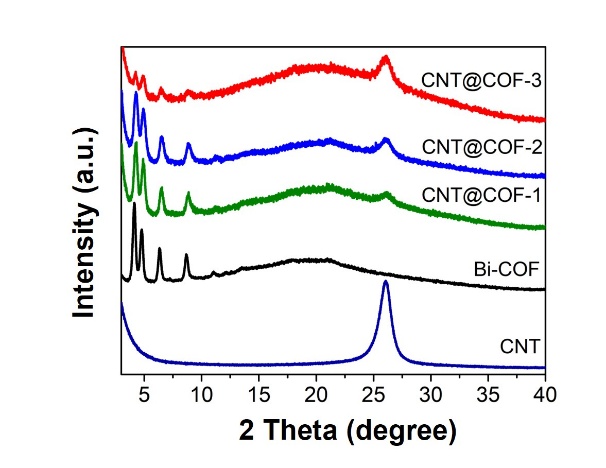


**Figure S3.** XRD patterns of CNT, Bi-COF, and CNT@COF composites.


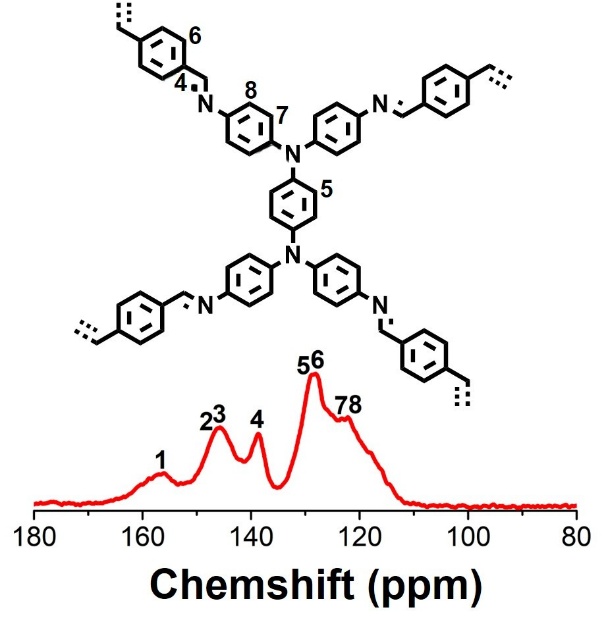


**Figure S4.** Solid State 13C CP-MAS NMR spectrum of Bi-COF.


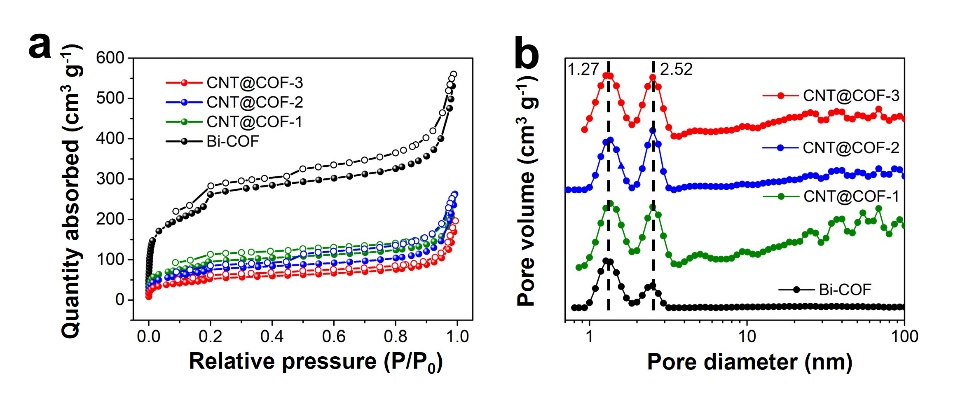


**Figure S5. (**a) N_2_ sorption isotherms and (b) Pore size distribution of Bi-COF and CNT@COF composites.


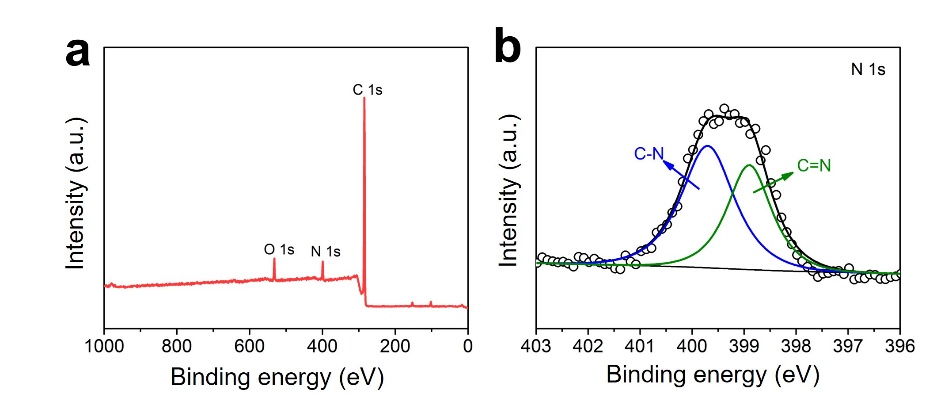


**Figure S6.** (a) Full survey XPS spectrum and (b) N 1s XPS spectra of the CNT@COF-3.


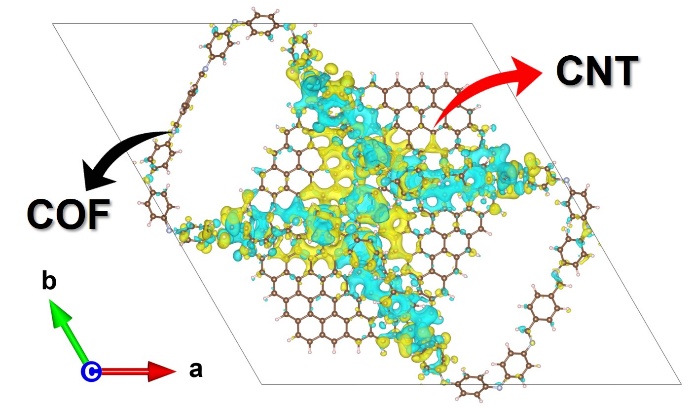


**Figure S7.** Vertical view of the charge density difference of CNT@COF.


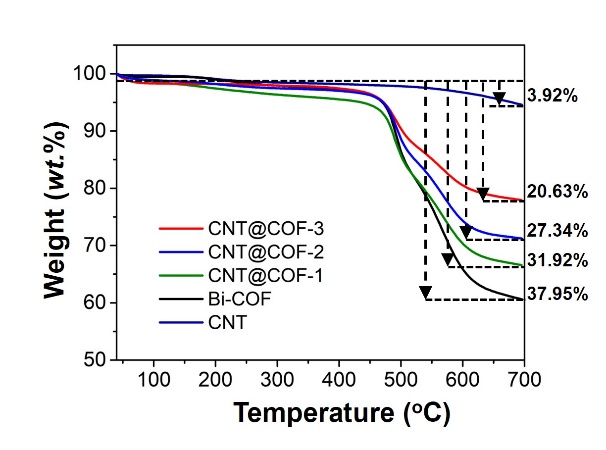


**Figure S8.** Thermal properties of CNT, Bi-COF and CNT@COF composites in the nitrogen atmosphere.


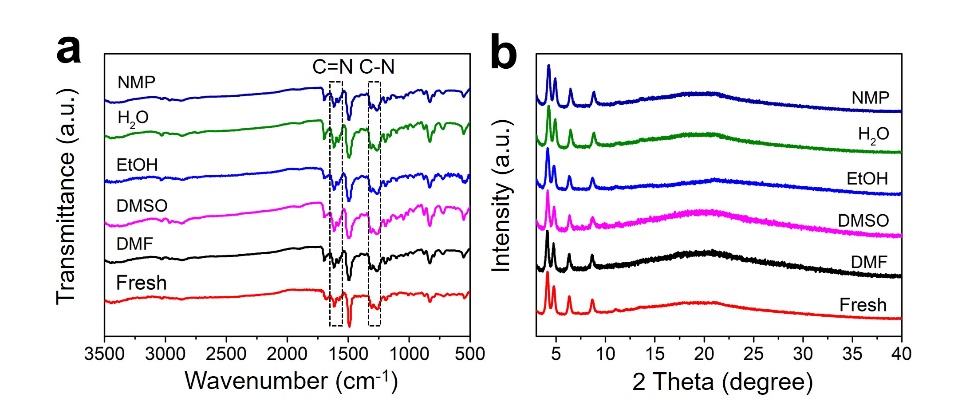


**Figure S9.** (a) FTIR spectra and (b) XRD patterns of Bi-COF after exposure to various organic solvents and water for 72 hours.


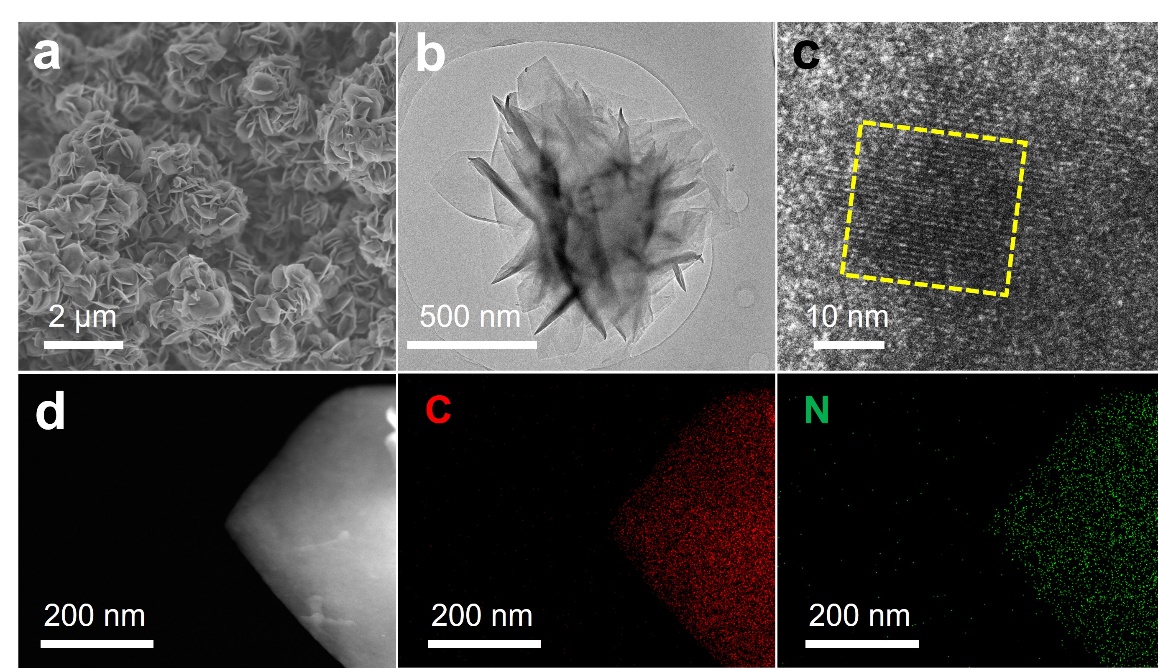


**Figure S10.** (a) SEM, (b) TEM and (c) HRTEM, and (d) energy dispersive spectroscopy (EDS) mappings of Bi-COF.


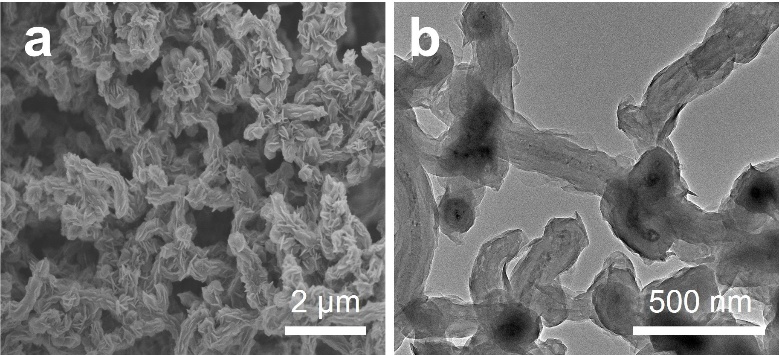


**Figure S11.** SEM and TEM images of CNT@COF-1.


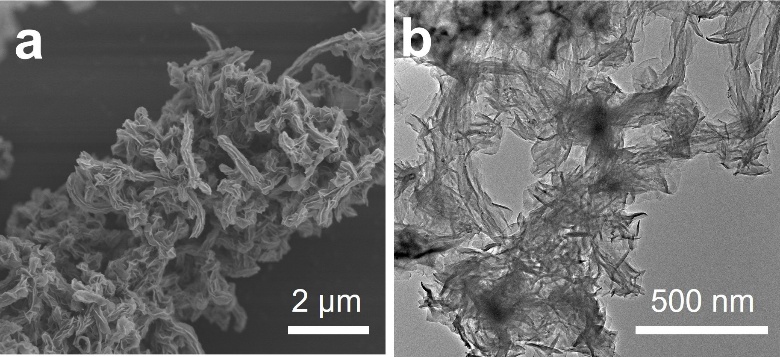


**Figure S12.** SEM and TEM images of CNT@COF-2.


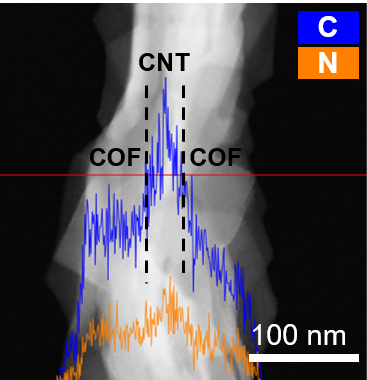


**Figure S13.** High-angle annular dark-field STEM and line-scan EDX analyses of CNT@COF-3.


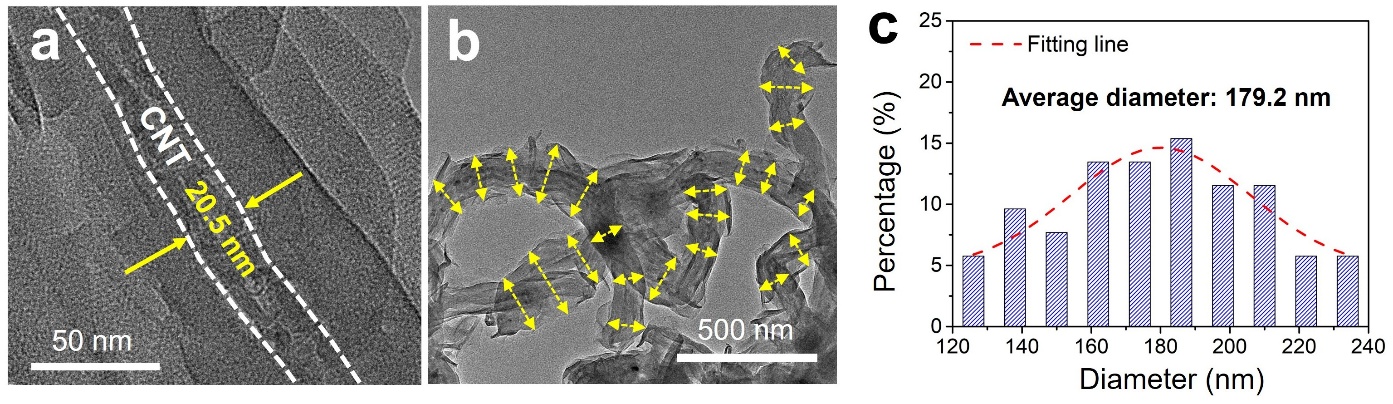


**Figure S14**. (a) The HRTEM image of CNT@COF-3. (b, c) TEM image of CNT@COF-3 and the related diameter size histogram. The average diameter size is indicated from the most symmetric histograms, which could be fitted by a log-normal distribution (The fits are shown as the red dotted line).


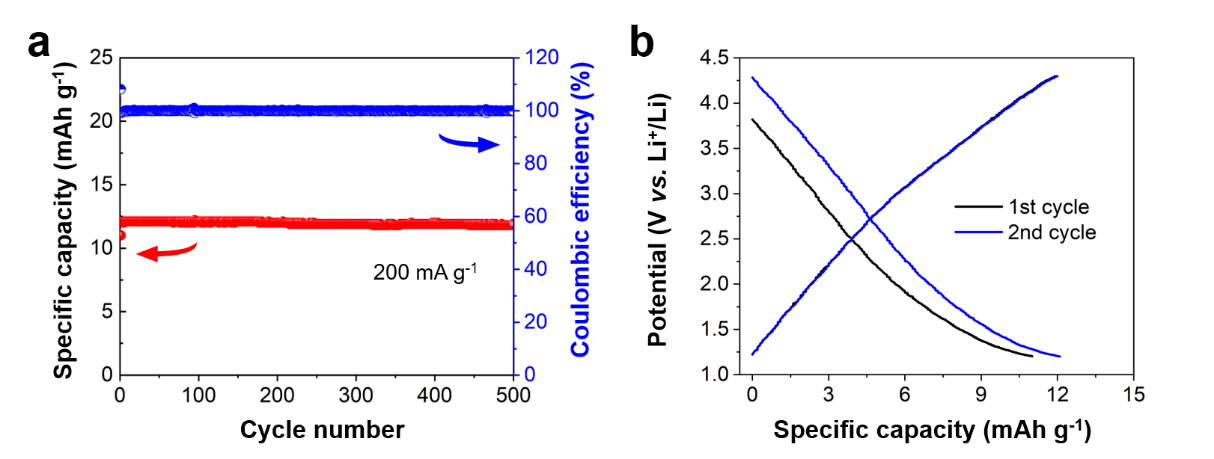


**Figure S15.** (a, b) Cycling performance of CNTs electrode at 200 mA g^−1^ for 500 cycles, and corresponding galvanostatic charge/discharge curves for the first two cycles.


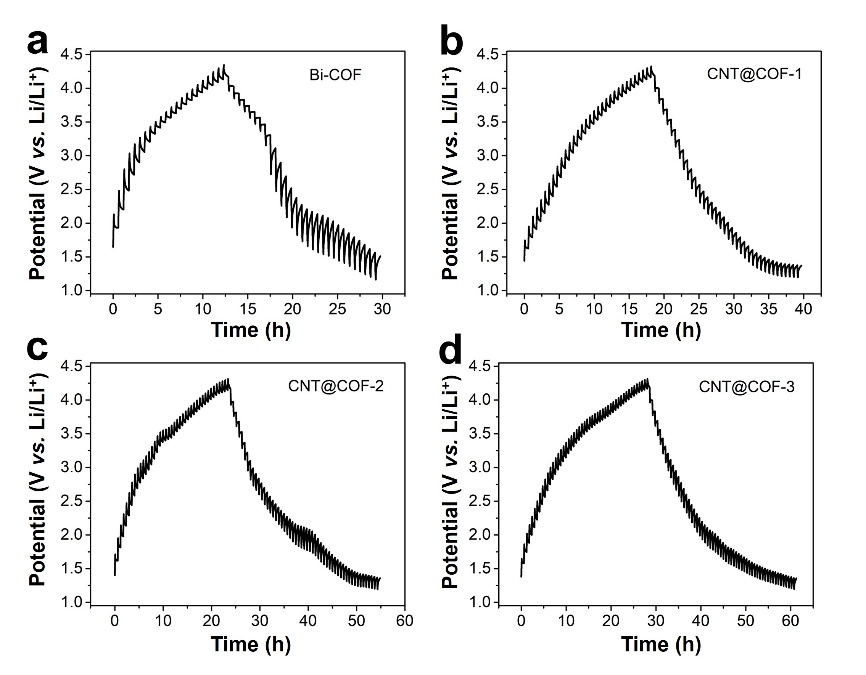


**Figure S16.** GITT curves of (a) Bi-COF, (b) CNT@COF-1, (c) CNT@COF-2, and (d) CNT@COF-3 at 20 mA g^-1^ during the discharge and charge processes.


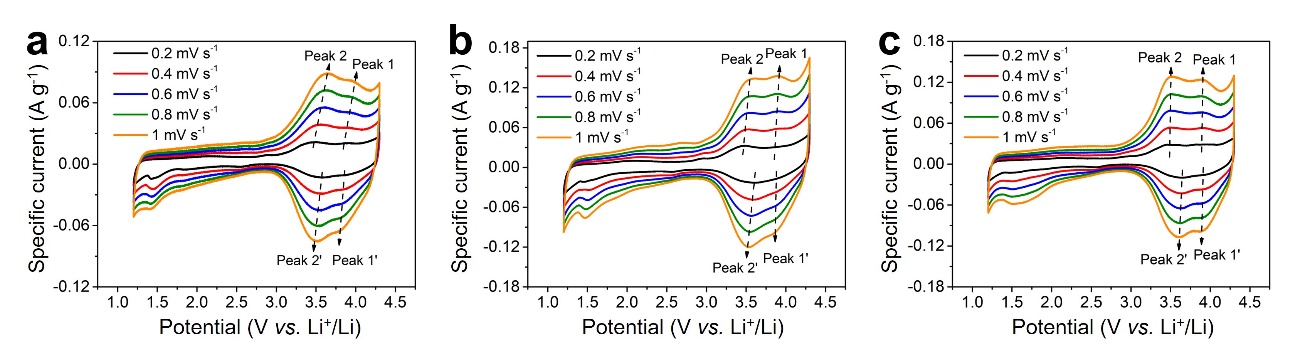


**Figure S17.** CV curves at different scan rates of (a) Bi-COF, (b) CNT@COF-1 and (c) CNT@COF-2.


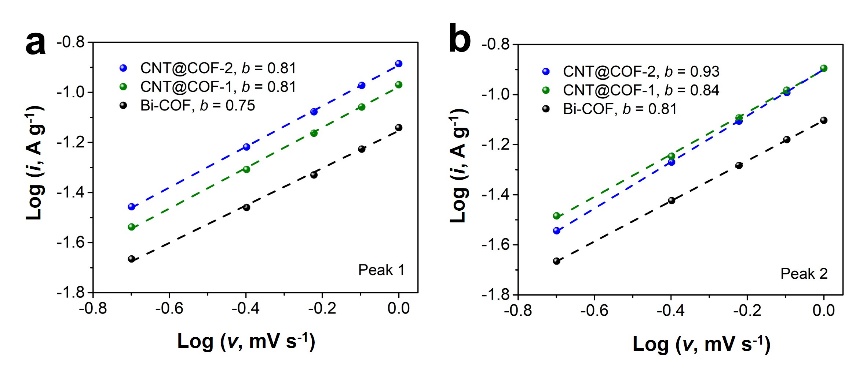


**Figure S18.** Log (*i*) versus log (*v*) plots of (a) Peak 1 and (b) Peak 2 of Bi-COF, CNT@COF-1, and CNT@COF-2.


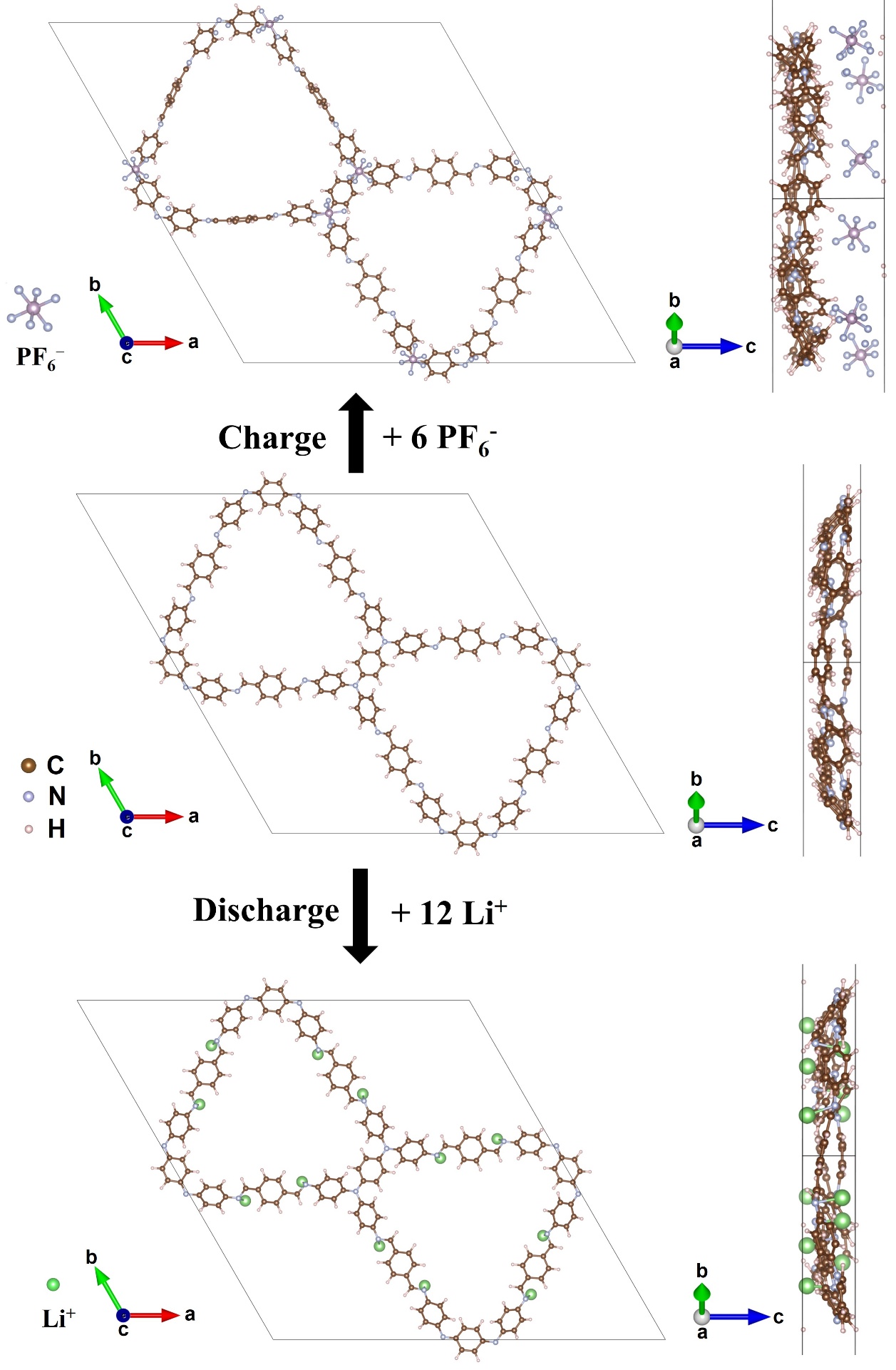


**Figure S19.** Structural evolution of a repeating unit within Bi-COF during charging and discharging process.

**Table S1.** Elemental analysis results of Bi-COF

| Element | C % | N % | H % |
| --- | --- | --- | --- |
| Theoretical value | 82.61 | 12.57 | 4.82 |
| Experimental value | 80.14 | 12.14 | 4.66 |

**Table S2.** Rate performance comparison of CNT@COF-3 with other reported COF-based electrodes electrode

| Electrode materials | Active sites | Capacity retention  (Current density_max_) | References |  |
| --- | --- | --- | --- | --- |
| CNT@COF-3 | C-N/C=N | 88.4% (5000 mA g^-1^) | This work | |
| DAPQ-COF50 | C=O | 86% (5000 mA g^-1^) | [1] | |
| 2D CCP-HATN@CNT | C=O/C=N | 81% (1000 mA g^-1^) | [2] | |
| PI-ECOF-1/rGO50 | C=O | 60% (1420 mA g^-1^) | [3] | |
| DAAQ-ECOF | C=O | 50% (3000 mA g^-1^) | [4] | |
| D_TP_-A_NDI_-COF@CNTs | C=O | 85% (1000 mA g^-1^) | [5] | |
| Tp-DANT-COF | C=O | 64% (2000 mA g^-1^) | [6] | |
| Tb-DANT-COF | C=O | 56% (2000 mA g^-1^) | [6] | |
| DAPO-TFB-COF | phenoxazine | 65% (800 mA g^-1^) | [7] | |
| DAPH-TFP | phenazine | 53% (3420 mA g^-1^) | [8] | |

**Table S3.** Cycling stability comparison of CNT@COF-3 with other reported COF-based electrodes electrode

| Electrode materials | Active sites | Capacity retention  (Current density) | References |
| --- | --- | --- | --- |
| CNT@COF | C-N/C=N | 83% after 5000 cycles  (5000 mA g^-1^) | This work |
| DAPQ-COF50 | C=O | 76% after 3000 cycles  (2000 mA g^-1^) | [1] |
| Tp-DANT-COF | C=O | 85% after 600 cycles  (1000 mA g^-1^) | [6] |
| DAPO-TpMe-COF | phenoxazine | 79% after 200 cycles  (100 mA g^-1^) | [7] |
| DAPH -TFP | phenoxazine | 31% after 500 cycles  (171 mA g^-1^) | [8] |
| E-TP-COF | C=O/C=N | 87.3% after 500 cycles  (200 mA g^-1^) | [9] |
| BFPPQ@CNT-50 | C=O/C=N | 86% after 600 cycles  (400 mA g^-1^) | [10] |
| BQ1-COF | C=O/C=N | 81% after 1000 cycles  (1540 mA g^-1^) | [11] |
| TAPT-NTCDA@CNT | C=O | 87.3% after 500 cycles  (1000 mA g^-1^) | [12] |
| NT-PICOF | C-N/C=O | 91% after 4000 cycles  (1000 mA g^-1^) | [13] |
| TPPDA-PICOF | C-N | 65% after 3000 cycles  (1000 mA g^-1^) | [14] |
| TPPDA-CuPorCOF | C-N | 86% after 3000 cycles  (1000 mA g^-1^) | [15] |
|  |  |  |  |
|  |  |  |  |
|  |  |  |  |
|  |  |  |  |

**References**

1. H. Gao, Q. Zhu, A. R. Neale, M. Bahri, X. Wang, H. Yang, L. Liu, R. Clowes, N. Browning, R. Sprick, M. Little, L. Hardwick, A. Cooper, *Adv. Energy Mater.* **2021**, *11*, 39.
2. S. Xu, G. Wang, B. P. Biswal, M. Addicoat, S. Paasch, W. Sheng, X. Zhuang, E. Brunner, T. Heine, R. Berger, X. Feng, *Angew. Chem. Int. Ed.* **2019**, *58*, 849.
3. Z. Wang, Y. Li, P. Liu, Q. Qi, F. Zhang, G. Lu, X. Zhao, X. Huang, *Nanoscale* **2019**, *11*, 5330.s
4. S. Wang, Q. Wang, P. Shao, Y. Han, X. Gao, L. Ma, S. Yuan, X. Ma, J. Zhou, X. Feng, B. Wang, *J. Am. Chem. Soc.* **2017**, *139*, 4258.
5. F. Xu, S. Jin, H. Zhong, D. Wu, X. Yang, X. Chen, H. Wei, R. Fu, D. Jiang, *Sci. Rep.* **2015**, *5*, 1.
6. D. Yang, Z. Yao, D. Wu, Y. Zhang, Z. Zhou, X. Bu, *J. Mater. Chem. A.* **2016**, *4*, 18621.
7. Z. Meng, Y. Zhang, M. Dong, Y. Zhang, F. Cui, T. Loh, Y. Jin, W. Zhang, H. Yang, Y. Du, *J. Mater. Chem. A.* **2021**, *9*, 10661.
8. E. Vitaku, C. Gannett, K. L. Carpenter, L. Shen, H. Abruña, W. Dichtel, *J. Am. Chem. Soc.* **2019**, *142*, 16.
9. G. Zhao, H. Li, Z. Gao, L. Xu, Z. Mei, S. Cai, T. Liu, X. Yang, H. Guo, X. Sun, *Adv. Funct. Mater.* **2021**, *31*, 29.
10. C. Jia, A. Duan, C. Liu, W. Z. Wang, S. X. Gan, Q. Y. Qi, Y. Li, X. Huang, X. Zhao, *Small* **2023**, e2300518.
11. M. Wu, Y. Zhao, B. Sun, Z. Sun, C. Li, Y. Han, L. Xu, Z. Ge, Y. Ren, M. Zhang, Q. Zhang, Y. Lu, W. Wang, Y. Ma, Y. Chen, *Nano Energy* **2020**, *70*.
12. K. Li, Y. Wang, B. Gao, X. Lv, Z. Si, H. G. Wang, *J. Colloid. Interface Sci.* **2021**, *601*, 446.
13. S. Gu, J. Chen, R. Hao, X. Chen, Z. Wang, I. Hussain, G. Liu, K. Liu, Q. Gan, Z. Li, H. Guo, Y. Li, H. Huang, K. Liao, K. Zhang, Z. Lu, *Chem. Eng. J.* **2023**, *454*.
14. S. Gu, R. Hao, J. Chen, X. Chen, K. Liu, I. Hussain, G. Liu, Z. Wang, Q. Gan, H. Guo, M. Li, K. Zhang, Z. Lu, *Mater. Chem. Front.* **2022**, *6*, 2545.
15. L. Gong, X. Yang, Y. Gao, G. Yang, Z. Yu, X. Fu, Y. Wang, D. Qi, Y. Bian, K. Wang, J. Jiang, *J. Mater. Chem. A.* **2022**, *10*, 16595.
